# Exhausted parents in Japan: Preliminary validation of the Japanese version of the Parental Burnout Assessment

**DOI:** 10.1002/cad.20371

**Published:** 2020-10-07

**Authors:** Kaichiro Furutani, Taishi Kawamoto, Maryam Alimardani, Ken'ichiro Nakashima

**Affiliations:** ^1^ Faculty of Business Administration Hokkai‐Ggakuen University 4‐1‐40 Asahimachi Toyohiraku Sapporo Hokkaido 062‐8605 Japan; ^2^ Department of Psychology Chubu University 1200, Matsumoto Kasugai Aichi 487‐8501 Japan; ^3^ Department of Cognitive Science and Artificial Intelligence Tilburg University Warandelaan 2 Tilburg Brabant 5037 AB Netherlands; ^4^ Graduate School of Humanities and Social Sciences Hiroshima University 1‐1‐1 Kagamiyama Higashi‐Hiroshima Hiroshima 739‐8524 Japan

**Keywords:** job burnout, parental burnout, parenting, perfectionism, questionnaire

## Abstract

We examined the factorial structure and validity of a Japanese version of the Parental Burnout Assessment, the PBA‐J, with 1,500 Japanese parents. The Parental Burnout Assessment measures burnout using four dimensions: exhaustion in one's parental role, contrast in parental self, feelings of being fed up, and emotional distancing. Confirmatory factor analysis on the PBA‐J supported a four‐factor model. Multiple‐group structural equation modeling with parent participants was supported for the factor‐loading invariance model. Mothers had higher parental burnout scores than fathers. We found moderate‐to‐strong correlation coefficients between the PBA‐J and the Parental Burnout Inventory (PBI‐J; the comparative burnout measure), and weak‐to‐moderate correlation coefficients between the PBA‐J and job burnout, neuroticism, co‐parenting disagreement, and family disorganization. The PBA‐J was correlated with parental perfectionism, particularly with concern over mistakes rather than sociodemographic variables. Overall, our findings provide initial evidence for the validity of the PBA‐J.

Parenting is fraught with difficulties worldwide. Some parents try to raise their children by holding them to high standards or avoiding parenting mistakes. Such efforts can lead to parental burnout, an emotional disorder. To assess parental burnout, Roskam, Raes, and Mikolajczak (2017) developed the Parental Burnout Inventory (PBI), which comprises three factors: exhaustion in one's parental role (EX), emotional distancing from one's children (ED), and personal accomplishment (PA).

Following the work of Roskam and colleagues, researchers evaluated the validity of the PBI in different countries (Kawamoto, Furutani, & Alimardani,2018; Van Bakel, Van Engen, & Peters, 2018). Kawamoto, Furutani, and Alimardani (2018) tested the construct validity of the Japanese version of the PBI with results indicating weak‐to‐moderate correlations between parental burnout and job burnout, thereby providing evidence for the PBI‐J's validity. Parental burnout is associated with individual differences, including neuroticism (Mikolajczak, Raes, Avalosse, & Roskam, 2018) and perfectionism (Kawamoto & Furutani, 2018). However, parental burnout is associated to a lesser extent with sociodemographic variables, such as the number of children or time spent at work (Kawamoto et al., 2018; Mikolajczak et al., 2018). Thus, investigating the mechanisms behind parental burnout is critical.

The PBI was developed based on the Maslach Burnout Inventory (MBI; Maslach & Jackson, 1981), which measures occupational burnout; thus, it uses the MBI's factor structure. It remains unclear whether the structure of the PBI that emerged from earlier studies is the best representation of parental burnout. In response to this limitation, a new measure, the Parental Burnout Assessment (PBA) was created (Roskam, Brianda, & Mikolajczak, 2018). The PBA includes four dimensions: EX; contrast with previous parental self (CO); feelings of being fed up with one's parental role (FU); and ED.

While the PBI and PBA are highly correlated and demonstrate consistency regarding relationships with other variables, Roskam et al. (2018) argue that the PBA has four advantages over the PBI. First, the PBI does not include CO as an essential parental burnout dimension, but the PBA does. Second, the PA dimension in the PBI assesses parental burnout indirectly; however, in the PBA, all 23 items are clearly formulated to measure parental burnout directly and precisely. Third, in the parental role, the loss of pleasure and fulfillment takes precedence over the loss of efficacy (Roskam et al., 2018). The PBI may be inappropriate because parental fulfillment and efficacy are both represented by a single factor (PA). Fourth, unlike the PBI, the PBA is a free assessment tool.

For the above reasons, the PBA can be considered a good candidate for assessing parental burnout. However, the questionnaire has thus far mainly been used among French‐, English‐, Chinese‐, and Finnish‐speaking parents (Aunola, Sorkkila, & Tolvanen, 2020; Cheng et al., 2020; Roskam et al., 2018); its validity and reliability in other cultural contexts and languages remains unknown. The possibility of cultural differences in the structure and expression of parental burnout is undeniable. Therefore, it is necessary to evaluate the validity and reliability of the PBA in different cultural contexts and languages. Consequently, for the present study, we chose to translate the PBA into Japanese and investigate its validity and reliability among Japanese parents.

## 1 THE CURRENT STUDY

Our primary goal was to create and validate a Japanese version of the PBA (PBA‐J); however, we set four sub‐goals. First, we tested whether the PBA‐J had a four‐factor structure identical to that of the original PBA. Additionally, as in previous studies (Aunola et al., 2020), factor invariance between mothers and fathers was tested.

Second, we examined gender effects of parental burnout in Japan, as mothers have been found to have higher burnout scores than fathers in Europe (Aunola et al., 2020; Roskam et al., 2018).

Third, we investigated the associations among the PBA‐J, PBI‐J, job burnout, depression, neuroticism, co‐parenting disagreement, and family disorganization. We explored the possibility of a moderate‐to‐strong correlation between the PBA and PBI (Roskam et al., 2018), and weak‐to‐moderate positive correlations for the PBA and job burnout (Brianda et al., 2020; Mikolajczak, Gross, Stinglhamber, Norberg, & Roskam, 2020). Moreover, we explored the possibility that parental burnout would be moderately positively associated with depressive symptoms (Kawamoto et al., 2018; Mikolajczak et al., 2020; Van Bakel et al., 2018). Previous studies reported weak‐to‐moderate correlations between the PBA and neuroticism, co‐parenting disagreement, and family disorganization (Gérain & Zech, 2018; Mikolajczak, Brianda, Avalosse, & Roskam, 2018; Roskam et al., 2018); thus, we explored whether the PBA‐J and those variables have weak‐to‐moderate positive correlations.

Fourth, following Kawamoto et al. (2018), we examined the influence of sociodemographic variables and perfectionism (personal standards [PS] and concern over mistakes [CM]) on parental burnout. Perfectionism is a personality trait comprising two dimensions: perfectionistic strivings and perfectionistic concerns (see Stoeber & Gaudreau, 2017; Stoeber & Otto, 2006). Perfectionistic strivings (here, PS) are personal standards of performance and a self‐oriented quest for perfection, while perfectionistic concerns (here, CM) are worries about making mistakes, fears of negative evaluation by others if one fails to be perfect, etc. PS had a weak negative correlation with parental burnout in the PBI‐J, while CM had a weak‐to‐moderate positive correlation (Kawamoto et al., 2018). Thus, we investigated the possibility that CM would have a stronger impact than PS on parental burnout in the PBA‐J. Moreover, in the PBI‐J, parental perfectionism had a stronger effect on parental burnout than did job perfectionism (Kawamoto et al., 2018). Thus, we examined the possibility that parental perfectionism would have more of an effect on parental burnout in the PBA‐J than job perfectionism would.

Incidentally, the literature reports inconsistent findings regarding the effects of sociodemographic variables on parental burnout. The PBI‐J was weakly negatively associated with sociodemographic variables such as parental age and number of children (Kawamoto et al., 2018). However, in Roskam et al. (2018) PBA study, parents who had a child with special needs, or had at least one child younger than five, or worked part time, had significantly higher PBA total scores. These variables were also included in Kawamoto et al. (2018). It is possible that this discrepancy may have been caused by differences in scales or cultures. Therefore, in this study, we also aimed to confirm the effects of sociodemographic variables.

## 2 METHOD

### 2.1 Participants

Participants (*N* = 1,500), who were recruited by a pooling company (Rakuten Insight), included parents living with at least one child in Japan. We used an approximately equal size allocation procedure for age and gender. Participant ages ranged from 20 to 59 years (*M* = 40.42; *SD* = 1.26). Participants provided written informed consent, and the ethics committee of Hiroshima University approved the study protocol.

### 2.2 Sociodemographic variables

In consideration of the Japanese family situation, we measured several demographic variables used in previous studies (Kawamoto et al., 2018; Roskam et al., 2018), including participants’ gender, age, number of children, age of children, marital status (married and living with marital partner; married but partner is transferred to a location far away from the family; married but living separately by choice; not married but living with a significant other or fiancé; not married and not living with a significant other or fiancé), level of education (junior high, high school, technical or 2‐year college, university, or graduate school), annual net income (1 = *less than 1 million yen* [about $10,000 USD] to 11 = *over 10 million yen* [about $100,000 USD]), type of work (full time or part time), and work hours per week. Table 1 summarizes participants’ characteristics.

**TABLE 1:** Descriptive statistics of the participants

	*N* or *M* [range]	
Child's age (years)	[0–36]	
Number of children	*M* = 1.62 [1–8]	
**Marital status**		
Living with a marital partner	1365	(91.00%)
Partner transferred to a location far away from family	37	(2.47%)
Living separately by choice	23	(1.53%)
Living with a significant other or fiancé (unmarried)	11	(0.73%)
Not living with a significant other or fiancé	64	(4.27%)
**Education level**		
9 years (junior high school)	32	(2.13%)
12 years (high school)	337	(22.47%)
12–16 years (technical or 2‐year college)	417	(27.80%)
16 years (undergraduates)	623	(41.53%)
>16 years	84	(5.60%)
Other	7	(0.47%)
**Work‐related variables**		
Annual net income^a^	*Me* = 7	
Working part time	241	(16.07%)
Working full time	920	(61.33%)
Full‐time homemaker	339	(22.60%)
Working hours per week	*M =* 33.10(*SD* = 19.48)	

*Note*. *N* = 1,500; *M*, mean; *Me*, median; *SD*, standard deviation.

^a^ 7 = 6 million to 7 million yen.

### 2.3 Measures

#### 2.3.1 Japanese version of the parental burnout assessment

The original PBA was translated into Japanese and back‐translated by a translation agency (Crimson Interactive, Inc., Japan) to ensure quality and content accuracy. We obtained permission to use and translate the PBA from Roskam, who developed the original version. An English–Japanese bilingual researcher compared and revised the questionnaires, paying attention to preserving the semantics and concepts of the original version. The original PBA comprises four subscales and 23 items: Exhaustion in Parental Role (EX: 9 items), Contrast with Previous Parental Self (CO: 6 items), Feelings of Being Fed Up (FU: 5 items), and Emotional Distancing (ED: 3 items). Items are rated on a 7‐point rating scale ranging from 0 = *never* to 3 = *a few times a month* to 6 = *every day*. In this sample, Cronbach's alphas were .95, .87, .93, .84 for the four subscales, respectively, and .98 for the total score.

#### 2.3.2 Japanese version of the parental burnout inventory

We included the 22‐item self‐report Parental Burnout Inventory‐Japanese version (PBI‐J; Kawamoto et al., 2018) for comparison. Mind Garden Inc., which holds the copyright to the PBI (1981, Christina Maslach & Susan E. Jackson; all rights reserved in all media), gave permission to alter the scale. The PBI comprises three subscales: Emotional Exhaustion (EX: 8 items), Emotional Distancing (ED: 8 items), and Personal Accomplishment (PA: 6 items, reverse scored). Items are rated on the same 7‐point scale as above. In this sample, Cronbach's alphas were .92, .89, and .85 for the three subscales, respectively, and .91 for the total score.

#### 2.3.3 Job burnout

We used the Japanese Burnout Inventory (JBI; Kubo, 1998, 2014; Tao & Kubo, 1996), which has been widely used in Japan, for instance by Kawamoto et al. (2018). This scale contains 20 items on three subscales: Emotional Exhaustion (EE: 7 items), Depersonalization (DP: 7 items), and Personal Accomplishment (PA: 6 items, reverse scored). Each item is rated a 7‐point scale ranging from 0 = *not at all* to 6 = e*very day*. Cronbach's alphas were .90, .89, and .88 for the three subscales, respectively, and .87 for the global score. For job burnout, only participants who were working part time or full time at the time of the study were asked to respond (*n* = 1,161).

#### 2.3.4 Depression

We used the Todai Health Index Depression Scale (THI‐D; Aoki, Suzuki, & Yanai, 1974: 10 items). Participants rated their feelings on a 3‐point scale: 1 = *no*; 2 = *neither yes or no*; and 3 = *yes*. Previous studies have reported good reliability (*α* = .91; Kawamoto et al., 2018) and validity (Kawada, Suzuki, Kubota, Ohnishi, & Satoh, 1999) for this scale. In the current sample, Cronbach's alpha was .91.

#### 2.3.5 Neuroticism

We used the neuroticism subscale of the 10‐item Personality Inventory‐Japanese version (TIPI; Gosling, Rentfrow, & Swann, 2003; TIPI‐J, Oshio, Abe, & Cutrone, 2012). The subscale includes two items rated on a 7‐point Likert‐type scale ranging from 1 = *strongly disagree* to 7 = *strongly agree*. In the current sample, Cronbach's alpha was .58.

#### 2.3.6 Co‐parenting disagreement

We used the Co‐parenting Agreement subscale (CRS; Feinberg, Brown, & Kan, 2012) of the Japanese version of the Co‐parenting Relationship Scale (CRS‐J: Takeishi, Nakamura, Kawaziri, Atogami, & Yoshizawa, 2017), which comprises four items rated on a 7‐point Likert‐type scale ranging from 0 = *not at all true for us* to 6 = *absolutely true for us*. Higher scores indicate greater co‐parenting disagreement. In the current sample, Cronbach's alpha was .83.

#### 2.3.7 Family disorganization

We used the Japanese version of the Confusion, Hubbub and Order Scale (CHAOS), a 15‐item scale measuring environmental confusion and disorganization in the family, such as high noise levels, crowding, and home traffic patterns (Matheny, Wachs, Ludwig, & Phillips, 1995; Matsumoto, 2012). Items were rated on a 5‐point Likert scale ranging from 1 *= not at all true for us* to 4 = *absolutely true for us*. Higher scores represent homes with greater chaos, disorganization, and time pressure. In the current study, Cronbach's alpha was .79.

#### 2.3.8 Parental and job perfectionism

We used the Japanese‐version Multidimensional Self‐oriented Perfectionism Scale (J‐MSPS; Sakurai & Ohtani, 1997), which is based on the Frost Multidimensional Perfectionism Scale (Frost, Marten, Lahart, & Rosenblate, 1990). The J‐MSPS includes four items that measure CM and five items that measure PS (Kawamoto & Furutani, 2018). Using both perfectionism subscales, we instructed participants to rate each item twice: when they described “parenting” and when they described “one's job.” Participants rated each item on a 6‐point scale ranging from 1 = *not at all* to 6 = *very much*. Cronbach's alphas for parental perfectionism were .87 and .89 for the two subscales and .91 for the global score. Cronbach's alphas for job perfectionism were .85 and .91 for the two subscales and .90 for the total score. For job perfectionism, only those who were working part time or full time at the time of the study were asked to respond.

### 2.4 Data analysis strategy

#### 2.4.1 Confirmatory factor analysis for all samples and by gender

First, to assess construct validity, we conducted a confirmatory factor analysis (CFA) for all samples. The analysis model included the four latent variables representing the concepts of EX, CO, FU, and ED, as well as their indicators, comprising 9, 6, 6, and 3 items, respectively. Analyses were conducted using maximum likelihood estimation. Reliability was estimated with Cronbach's alpha coefficients (*α*). Next, we tested the four‐factor structure model by gender. Several goodness‐of‐fit indices were used to determine the acceptability of the models. In addition to the chi‐square index, the comparative fit index (CFI), root mean square error of approximation (RMSEA), standardized root mean square residual (SRMR), and Tucker–Lewis index (TLI) were used to evaluate model fit, similar to previous studies (Roskam et al., 2017, 2018). A CFI and TRI of .90 or larger are acceptable, while values higher than .95 indicate a better fit to the data. RMSEA and SRMR should be less than or equal to .08; values less than or equal to .06 are preferable (Hu & Bentler, 1999).

#### 2.4.2 Gender differences in model structure by multiple‐group structural equation modeling

To test the measurement invariance between mothers and fathers of the four‐factor structure model of the PBA‐J, we first conducted multiple‐group structural equation modeling (MGSEM). If the configural invariant model provided acceptable fit indices to the data, it would suggest that the four‐factor structure of the PBA‐J was consistent across genders. Second, we tested the factor‐loading invariance model in which all factor loadings in the model were constrained to be equal using MGSEM. We calculated the fit index and checked whether the previously mentioned criterion fit index was accepted. Furthermore, we identified changes in several goodness‐of‐fit indices for the configural invariance model and factor‐loading invariance model. The difference of chi‐square (Satorra & Bentler, 2001) between the configural invariance model and factor‐loading invariance model was significant. However, when the sample size exceeds 300, and if the changes of the model fit indices (the configural invariance model's fit index and factor‐loading invariance model's fit index) are ΔCFI ≤ .010, ΔRMSEA ≤ .015, and ΔSRMR ≤ .030, then the factor‐loading invariance across groups is consistent (Chen, 2007). Thus, we adopted these criteria. Third, we tested the intercept‐loading invariance model, which constrained all intercepts to be consistent between genders using MGSEM. We calculated the fit index and checked whether the previously mentioned criterion fit index was accepted. Next, we adopted the criteria that if the changes of model fit indices (the factor‐loading invariance model's fit index and intercept‐loading invariance model's fit index) are ΔCFI ≤ .010, ΔRMSEA ≤ .015, and ΔSRMR ≤ .015, then the intercept‐loading invariance across groups is consistent (Chen, 2007); we checked the changes of model fit indices.

#### 2.4.3 Gender differences in PBA‐J

We examined the effect of gender on the total PBA‐J score and its subscales (EX, CO, FU, and ED). First, we conducted an analysis of variance (ANOVA) with gender as a fixed factor and total PBA‐J score as the dependent variable. Second, to control for the Type I error rate, we conducted a multivariate analysis of variance (MANOVA) with gender as a fixed factor and PBA‐J subscales as the dependent variables.

#### 2.4.4 Criterion‐related validity of PBA‐J

To investigate criterion‐related validity, we examined the associations among the PBA‐J and PBI‐J, job burnout, depression, neuroticism, co‐parenting disagreement, and family disorganization. We computed Pearson's correlation coefficients between the sum of the PBA‐J total and subscale scores, job burnout total and subscale scores, and scores for depression, neuroticism, co‐parenting disagreement, and family disorganization.

#### 2.4.5 Relationships between sociodemographic variables, perfectionism, and parental burnout

Similar to Kawamoto et al. (2018), to examine the influence of sociodemographic variables and perfectionism on parental burnout, we computed correlation coefficients between the total PBA‐J score and the mean scores of our continuous variables (i.e., parental and job perfectionism, parental and job PS, and parental and job CM). Moreover, to explore the relationships among the PBA‐J, sociodemographic variables, and perfectionism in detail, we conducted hierarchical multiple regression analysis; sociodemographic variables were entered into step 1, and parental PS, parental CM, job PS, and job CM variables were entered into step 2. This analysis included 1,161 people, excluding those who did not respond to the job perfectionism scale (*n* = 339).

We used Mplus 8.4 (Muthén & Muthén, 2017) for the CFA and MGSEM. For all other analyses, we used SPSS version 25.0.

## 3 RESULTS

### 3.1 Confirmatory factor analysis and reliability of the PBA‐J

Table 2 shows the factor loadings in CFA, mean, *SD*, skewness, and kurtosis for each PBA‐J item. The fit index of the four‐factor model for PBA‐J was acceptable (total sample: CFI = .93, TLI = .92, RMSEA = .085, SRMR = .037, *χ* ^2^(224) = 966.15, *p* < .001; father and mother data is presented in Table 3). The correlations between the mean scores of the four factors ranged from .80 to .89. The fit indices of the MGSEM to test the configural invariance model, factor‐loading invariance model, and intercept‐loading invariance model of the PBA‐J are presented in Table 3. These models were acceptable. Next, we examined the changes of model fit indices between the configural invariance model and factor‐loading invariance model. The chi‐square difference between these models was significant; however, the changes of the other fit indices (ΔCFI = .006, ΔRMSEA = .001, and ΔSRMR = .01) met the criteria recommended by Chen (2007). Therefore, the factor‐loading invariance model was supported. Next, we examined the changes of model fit indices between the factor‐loading invariance model and intercept‐loading invariance model. The chi‐square difference between these models was significant. Additionally, the differences for RMSEA and SRMR were acceptable (ΔRMSEA = .002, ΔSRMR = .003), but the difference for CFI was .012, and this change did not meet Chen's criterion. Therefore, the intercept‐loading invariance model was not supported.

**TABLE 2:** Factor loadings from CFA; mean, standard deviation, skewness, and kurtosis for each PBA‐J item

Items	EX	CO	FU	ED	*M*	*SD*	Skewness	Kurtosis
EX1	.83				1.08	1.68	1.57	1.40
EX2	.87				0.93	1.48	1.74	2.32
EX3	.87				1.05	1.63	1.61	1.60
EX4	.84				0.96	1.50	1.70	2.14
EX5	.83				1.00	1.63	1.70	1.90
EX6	.83				0.68	1.31	2.20	4.44
EX7	.83				0.75	1.39	2.12	3.93
EX8	.79				0.94	1.52	1.69	1.96
EX9	.72				1.07	1.61	1.57	1.54
CO1		.83			1.05	1.62	1.61	1.61
CO2		.83			0.82	1.42	1.89	2.83
CO3		.83			1.11	1.63	1.55	1.46
CO4		.84			0.92	1.57	1.77	2.21
CO5		.82			1.14	1.70	1.50	1.23
CO6		.83			0.89	1.49	1.80	2.41
FU1			.89		0.58	1.27	2.39	5.17
FU2			.88		1.12	1.64	1.51	1.33
FU3			.88		1.00	1.61	1.71	2.01
FU4			.76		0.71	1.36	2.06	3.60
FU5			.85		0.60	1.30	2.37	4.99
ED1				.67	1.21	1.68	1.43	1.10
ED2				.81	0.91	1.54	1.77	2.23
ED3				.86	1.17	1.70	1.47	1.15

*Note*. PBA‐J, Japanese version of Parental Burnout Assessment; EX, Exhaustion in One's Parental Role; CO, Contrast with Previous Parental Self; FU, Feelings of Being Fed up with One's Parental Role; ED, Emotional Distancing from One's Children. Each item's number corresponds to the item number from the original questionnaire by Roskam et al. (2018).

**TABLE 3:** Goodness‐of‐fit indices for the models of the Japanese Parental Burnout Assessment factor structure

Model	df	*χ* ^2^	*p*	CFI	TLI	RMSEA	SRMR	95% *CI*	Δdf	Δ*χ* ^2^	*p*	ΔRMSEA	ΔSRMR
Father	224	443.04	**	.962	.957	.036	.026	[.031–.041]					
Mother	224	799.14	**	.905	.893	.058	.047	[.054–.063]					
Configural invariance model	448	1177.26	**	.938	.930	.047	.038	[.043–.050]					
Factor‐loading invariance model	471	1274.94	**	.932	.927	.048	.048	[.045–.051]	23	121.55	**	.001	.010
Intercept invariance model	494	1430.52	**	.920	.918	.050	.051	[.047–.053]	23	333.36	**	.002	.003

*Note*. *CI*, confidence Interval. ** *p* < .01. Δ*χ* ^2^ were calculated based on Satorra and Bentler (2001).

### 3.2 Gender differences in PBA‐J scores

Table 4 presents the means and *SD* of PBI‐J scores for the total sample and mother and father subsamples. Mothers had significantly higher PBA‐J total scores than fathers. Additionally, the MANOVA with the four factors for burnout showed that parental burnout was, on average, higher among mothers than fathers (Wilks’ *λ* = .954, *F*(4, 1,495) = 18.181, *p* < .01). Follow‐up univariate tests revealed that compared to fathers, mothers had significantly higher EX and CO.

**TABLE 4:** Means, standard deviations, and analysis of variance results for PBA‐J by parental role

		All Sample	Fathers	Mothers			
Measure		(*N* = 1500)	(*n* = 752)	(*n* = 748)	*F*	Partialη^2^	*CI*
PBA‐J total	*M*	21.70	19.57	23.82	8.12^**^	.005	[.001, .015]
	*SD*	28.96	28.39	29.39			
EX	*M*	9.41	8.02	10.79	19.44^**^	.013	[.004, .026]
	*SD*	12.26	11.44	12.88			
CO	*M*	5.69	5.23	6.15	4.88^*^	.003	[.000, .011]
	*SD*	8.09	7.85	8.30			
FU	*M*	3.94	3.83	4.05	0.48	.000	[.000, .005]
	*SD*	6.17	6.20	6.15			
ED	*M*	2.66	2.49	2.82	2.77	.002	[.000, .009]
	*SD*	3.92	3.85	3.98			

*Note*. PBA‐J, Japanese version of the Parental Burnout Assessment; EX, Exhaustion in Parental Role; CO, Contrast with Previous Parental Self; FU, Feelings of Being Fed Up; ED, Emotional Distancing. ** *p* < .01, * *p* < .05.

### 3.3 Relationships between PBA‐J, PBI‐J, and JBI

Pearson's correlations coefficient among the PBA‐J scores (total score, EX, CO, PA, and FU), PBI‐J scores (total score, EX, ED, and PA), JBI scores (total score, EE, DP, and PA), depression, neuroticism, co‐parenting disagreement, and family disorganization are presented in Table 5 in Supporting Information. We found moderate to significantly strong positive correlations between the four PBA‐J scores and PBI‐J scores except PA. Weak‐to‐moderate positive correlations were observed between PBA‐J scores and JBI total score, EE, DP, and depression. PBA‐J and PA in JBI scores had significantly negative correlations. The correlations between PBA‐J and neuroticism and co‐parenting disagreement were low, and correlations with family disorganization were weak‐to‐moderate.

**TABLE 5:** Correlation coefficients, means, standard deviations for the PBA‐J, PBI‐J, JBI, depression, neuroticism, co‐parenting disagreement, family disorganization, and perfectionism variables

	PBA‐J total	PBA‐J EX	PBA‐J CO	PBA‐J FU	PBA‐J ED	*M*	*SD*
PBA‐J total	–					21.70	28.96
PBA‐J EX	.97					9.41	12.26
PBA‐J CO	.95	.89	–			5.69	8.09
PBA‐J FU	.94	.89	.86			3.94	6.17
PBA‐J ED	.90	.84	.80	.84	–	2.66	3.92
PBI‐J Total	.77	.76	.73	.72	.71	39.56	20.73
PBI‐J EE	.71	.67	.68	.67	.72	9.70	10.31
PBI‐J ED	.76	.78	.70	.70	.63	11.42	11.01
PBI‐J PA (reversed)	.03	.01	.03	.03	.04	18.44	9.63
JBI Total	.30	.30	.30	.29	.26	45.64	17.02
JBI EE	.37	.38	.36	.34	.33	11.86	7.84
JBI DP	.42	.41	.41	.41	.39	10.81	8.67
JBI PA (reversed)	−.17	−.17	−.16	−.16	−.17	22.97	8.35
Depression	.43	.42	.44	.40	.35	17.94	5.75
Neuroticism	.32	.33	.32	.27	.21	8.08	2.49
Co‐parenting disagreement	.29	.28	.30	.27	.26	11.73	4.41
Family disorganization	.48	.46	.48	.44	.38	30.12	6.80
Parental perfectionism total	.35	.32	.34	.36	.32	19.43	9.02
Parental perfectionism PS	.24	.22	.24	.24	.24	11.96	5.80
Parental perfectionism CM	.43	.40	.42	.44	.40	7.47	4.08
Job perfectionism total	.27	.26	.27	.25	.23	25.04	9.67
Job perfectionism PS	.13	.13	.14	.12	.10	15.57	6.31
Job perfectionism CM	.38	.37	.38	.36	.35	9.47	4.60

*Note*. *N* = 1,500 (when analysis of JBI or job perfectionism, *N* = 1,161). PBA‐J, Japanese version of the Parental Burnout Assessment; EX, Exhaustion in One's Parental Role; CO, Contrast with Previous Parental Self; FU, Feelings of Being Fed up with One's Parental Role; ED, Emotional Distancing from one's children; PBI‐J, Japanese version of Parental Burnout Inventory; EE, Emotional Exhaustion; PA, Personal Accomplishment; JBI, Japanese Burnout Inventory (job burnout); DP, Depersonalization; PS, Personal Standards; CM, Concern over Mistakes.

*r* ≥ .06 are significant at *p* < .05 and *r* > .07 results in *p* < .01.

### 3.4 Relationships between perfectionism, sociodemographic variables, and PBA‐J

Parental/job perfectionism, parental/job PS, and parental/job CM had weak positive relationships with PBA‐J scores (Table 5). Parental CM and PBA‐J scores showed a moderate positive relationship. Moreover, perfectionism and parental PS had a weak positive relationship with PBA‐J scores. To examine the effect of sociodemographic variables and perfectionism on PBA‐J, we conducted a hierarchical multiple regression analysis (Table 6). In step 1, analysis showed that the sociodemographic variables *having a child with special needs* and *living separately by choice* were associated with PBA‐J total scores, explaining minimal variance for PBA‐J (4.0%). In step 2, perfectionism explained more variance for parental burnout (23.0%). Parental and job CM were significantly linked to the PBA‐J total score; specifically, the parental CM effect was stronger than job CM. In addition to the sociodemographic variables *having a child with special needs* and *living separately by choice*, gender significantly and positively predicted PBA‐J.

**TABLE 6:** Hierarchical multiple liner regression analysis summary for sociodemographic variables and perfectionism to predict PBA‐J

Predictor variables	Step1	Step2
Sociodemographic variables		
Gender (0 = father, 1 = mother)	.05	.08^**^
Age of parent	−.04	.00
Number of children	.00	.00
Having younger children (<5 years old)	.05	.07
Having a child with special needs (0 = No, 1 = Yes)	.12^**^	.09^**^
Education level	.00	−.01
Working part time^a^	.02	.00
Work hours per week	.00	.01
Household income	.00	.00
Partner transferred to a location far away from family^b^	.00	−.02
Living separately by choice^b^	.10^**^	.07^**^
Living with a significant other or fiancé^b^	.03	.02
Not living with a significant other or fiancé^b^	−.03	−.01
Perfectionism		
Parental PS		−.07
Parental CM		.39^**^
Job PS		−.06
Job CM		.21^**^
*R* ^2^	.04^**^	.27^**^
Δ*R* ^2^	.04^**^	.23^**^

*Note. N* = 1,161. Standardized regression coefficients reported for predictor. PBA‐J, Japanese version of Parental Burnout Assessment; PS, Personal Standards; CM, Concern over Mistakes.

^a^ Reference category is working full time.

^b^ Reference category is living with a marital partner.

** *p* < .01.

## 4 DISCUSSION

This study examined the characteristics of parental burnout in Japan by successfully testing the validity of PBA‐J. First, similar to studies of French‐, English‐, Chinese‐, and Finnish‐speaking parents, our results provide evidence for the validity of the PBA‐J and its four‐factor structure. However, it should be noted that the current study's results supported a factor‐loading invariance model. Second, mothers were more likely to suffer parental burnout than fathers. These results were consistent with previous findings that parental burnout symptoms are, to some extent, dependent on the parent's gender (Roskam et al., 2018; Sorkkila & Aunola, 2020). Third, we found weak‐to‐moderate correlations between the PBA‐J, job burnout, and depression. These findings were similar to those of prior studies (e.g., Mikolajczak et al., 2020; Roskam et al., 2018). Fourth, little association emerged between the PBA‐J and sociodemographic variables with the exception of gender, whether the parent had a child with special needs, and whether parents lived separately by choice. However, associations emerged between PBA‐J and neuroticism, co‐parenting disagreement, family disorganization, parental perfectionism, parental CM, and job CM. These results are consistent with previous studies (Kawamoto et al., 2018; Roskam et al., 2018) and indicate that some aspects of parental burnout are common across Europe and Japan.

### 4.1 Structure of the PBA‐J

We tested whether the factor structure of the PBA‐J was identical to the four‐factor structure of the original PBA. The results showed that, similar to Roskam et al. (2018), the PBA‐J is composed of four factors: EX, CO, FU, and ED. Correlation analysis results showed that the four parental burnout subscales were strongly associated with each other. From these findings, and in the context of Japanese culture, it can be concluded that the PBA‐J has a four‐factor structure consisting of four positively correlated factors. Additionally, factor invariance between mothers and fathers was tested using MGSEM, revealing that the four‐factor model of the PBA‐J had acceptable measurement invariance at the scale and factor‐loading levels between genders. However, we did not confirm the equivalence at the intercept level of the PBA‐J. In the future, it will be necessary to explore item‐level analysis to identify which item intercepts are non‐invariant between genders.

### 4.2 Gender differences in the PBA‐J

In the present study, gender associated to PBA‐J in the multiple regression analysis, and mothers’ PBA‐J total scores were higher than fathers’ scores. These results were similar to the findings of Roskam et al. (2018) and Aunola et al. (2020). However, there are at least two problems with interpreting these differences simply to mean that mothers suffer more from burnout than fathers. First, there are no clinical cut‐offs defined for the PBA‐J, making it difficult to verify when parental burnout had really started. Second, it cannot be determined whether these differences are related to gender or parental roles; to do so, one would need to look at the PBA‐J responses of same sex parents. Only if we were to find no differences in these parental constellations would we be able to interpret these differences as gender related. Otherwise they should be regarded as parental‐role‐specific differences.

### 4.3 Relationship of PBA‐J and PBI‐J

Correlation analysis showed that the results were almost in line with the results of previous research, although one point of interest was the relationship between the PBA and PA in the PBI. While Roskam et al. (2018) found a significant weak association between the PBA and PA in the PBI, the present study found no association between them. Also, confirming the findings of previous studies of the PBI, a weak‐to‐moderate association was found between ED, EE, and PA (Van Bakel et al., 2018; Roskam et al, 2017). However, these results of were not replicated by Kawamoto et al. (2018), who showed that only ED and EE, not PA, were correlated in Japanese samples. Therefore, the independence of PA and other measures of burnout are currently unique features of the Japanese population and should be further considered in future studies.

### 4.4 Perfectionism and PBA‐J

Parental perfectionism more strongly influenced parental burnout than job perfectionism did, and CM influenced parental burnout more strongly than PS did. The PBA‐J, like the PBI‐J, was associated with parental perfectionism, particularly parental perfectionistic concerns. Previous research examined the relationship between PBA and self‐oriented perfectionism, the relationship between PBA and socially prescribed perfectionism, and their interaction (Sorkkila & Aunola, 2020). These studies indicated that perfectionism directly affects adaptive and maladaptive variables (such as burnout), along with indirectly affecting other variables. These indirect variables include the tendency to use all‐or‐nothing thinking, intolerance for uncertainty, overgeneralizing negative events, ruminating about past failures, and lack of social support (Flett, Coulter, Hewitt, & Nepon, 2011; Kawamoto & Furutani, 2018; Smith et al., 2010). Therefore, researchers should consider other factors, including socially prescribed perfectionism, interactions between different kinds of perfectionism, and potential mediators to further assess the mechanisms behind parental burnout. Additionally, since perfectionism is a risk factor for parental burnout (see Kawamoto et al., 2018), such studies will provide useful information for psychotherapy, such as cognitive behavioral therapy, focused on perfectionism (Lombardo, 2014) as a cause of burnout.

### 4.5 Sociodemographic variables and PBA‐J

Our findings indicated that *having a child with special needs* had a weak positive effect on PBA‐J. This finding supported the results of Roskam et al. (2018). The parents of children with developmental disabilities and mental health problems have more negative agitation and physical problems than parents of children without these issues (Feizi, Najmi, Salesi, Chorami, & Hoveidafar, 2014). This suggests that parents raising a child with special needs have a higher degree of burnout. Additionally, our findings indicated that *living separately by choice* was a predictor of PBA‐J. This may be due to having less partner support and the burden of parenting alone. However, there was no effect on PBA‐J from the variable *not living with a significant other or fiancé*. Therefore, it is important to keep in mind networks of non‐partners. To clarify this difference in effect, it would be worth focusing on the influence of social networks other than partners (Nelson, 1995). Furthermore, the results of this study suggest that culture and sample may affect the influence of sociodemographic variables; thus, these factors should be carefully considered in future studies.

### 4.6 Limitations and future directions

Future studies should further investigate validity of PBA‐J. The correlation of PBA‐J and PBI‐J could be interpreted as a convergent indication of construct validity; however, future studies should seek to provide evidence for discriminant and criterion validity, too. Criterion validity, in particular, would require investigating the prediction of clinically diagnosed burnout symptoms, along with other real life consequences of burnout. Future studies should examine which PBA‐J subscales are affected by predictor variables to deepen understanding of PBA‐J and parental burnout. Additionally, clinical cut‐off scores should be defined in order to identify when burnout really starts. As Roskam et al. (2018) argued, future studies using objective external criteria are needed. Finally, the characteristics of the parenting environment in Japan and the influence of unique cultural factors (e.g., Japanese women's difficulties in restarting a career after having a child) on the PBA‐J should also be explored, to further expand our understanding of parental burnout.

## 5 CONCLUSION

The present research provides preliminary evidence regarding the validity and reliability of the PBA‐J. Moreover, our results revealed that parental perfectionistic concerns can be a risk factor for parental burnout.

## Supporting information

Supporting Information
