# Supplementary material for: Exhausted parents in Japan: Preliminary validation of the Japanese version of the Parental Burnout Assessment
Source: New Dir Child Adolesc Dev. 2020 Oct 7;2020(174):33–49. doi: 10.1002/cad.20371 (PMC7821145; doi:10.1002/cad.20371)
Supplement: Supplementary file 1 — Supporting Information [file CAD-2020-33-s001.pdf]

Items of PBA-J：PBA 日本語版（PBA-J）

教示文

　親にとって子どもは、充足感と喜びをもたらしてくれる存在として重要です。それと同時に、一部の親にとっては、子どもは疲労の原因ともなります。（これは、矛盾ではありません。自己実現と疲労とは共存する場合がありますし、自分の子どもを愛しながら、同時に親としての役割に疲れ果ててしまうこともありえます）。以下のアンケートは、親として経験する可能性がある「情緒的な疲労」に関するものです。ご自分の気持ちに一番近い回答を、選択してください。以下の各文について、そうした感情を全く経験したことがなければ、「全くない」を選んでください。そうした感情を体験したことがあれば、どの程度の頻度でそう感じるか、「年に数回以下」から「毎日」までのいずれかを選んでお答えください。

0：全くない
1：年に数回以下
2：月に１回
3：月に数回
4：週に１回
5：週に数回
6：毎日

EX1. 親としての自分の役割にすっかり疲れ果てている
EX2. 親として本当に疲れ果ててしまっている感覚がある
EX3. 親としての自分の役割に疲れてしまっているので、寝ても疲れが取れないように思える
EX4. 朝起きて、また１日子供の相手をしないといけない時、始める前から疲れ果ててしまうように感じる
EX5. 自分の子供のために、あれもこれもしないといけないと考えるだけで、くたくたになってしまう
EX6. 自分の子供の世話をするエネルギーが全くない
EX7. 親としての役割は、私の能力を使い果たしてしまう
EX8. 自分の子供の世話を、まるで自動操縦装置のようにやってしまっていると感じることがある
EX9. 親としての自分の役割となると、最低限のことさえやっていれば良いという状態になっている
CO1. 以前のような良い父親または母親ではなくなってしまっていると思う
CO2. 自分は以前のような親ではなくなってしまったと内心思う
CO3. 自分はひどい親になってしまったと、恥ずかしく思う
CO4. 親としての自分に、もう誇りを持てない
CO5. 自分の子供の相手をしている時、もう自分が自分でないように感じる
CO6. 父親または母親としての、自分の方向性を見失っているように感じる
FU1. 父親または母親としての自分の役割には、もう耐えられない
FU2. もうこれ以上、親であることに耐えられない
FU3. もう、親などやっていられないと感じる
FU4. 親という仕事をこなせないと感じる
FU5. 自分の子供と一緒にいることが楽しくない
ED1. 親として子供にしなければいけないことはしているが、それ以上のことはしない
ED2. 毎日の決まった仕事（車で送る、寝かしつけ、食事）以外に、もはや子供のために努力することができない
ED3. 自分の子供に対して、もはや愛情を示すことができない

EX: Exhaustion in One's Parental Role:（親役割についての）情緒的消耗感
CO: Contrast with Previous Parental Self:（親である）過去の自分との対比
FU: Feelings of Being Fed up with One's Parental Role:（親役割に対する）うんざり感
ED: Emotional Distancing from one's children:（子どもとの）感情的距離

各項目番号はPBA（Roskam et al.,2018)と同様である。
